# Case Series: Computed Tomography Features of Extraskeletal Osteosarcoma in Six Dogs

**DOI:** 10.3390/vetsci11060282

**Published:** 2024-06-20

**Authors:** Jeongyun Jeong, Minjoo Kim, Sung-Soo Kim, Hyunju Hwang, Joohyun Jung, Noh-Won Park, Jaehwan Kim, Kidong Eom

**Affiliations:** 1 Department of Veterinary Medical Imaging, College of Veterinary Medicine, Konkuk University, Seoul 05029, Republic of Korea; jju9510@konkuk.ac.kr; 2 Shine Animal Medical Center, Seoul 05550, Republic of Korea; 3 VIP Animal Medical Center, Seoul 02830, Republic of Korea; 4 Ilsan Animal Medical Center, Ilsan 10368, Republic of Korea; 5 Nowon N Animal Medical Center, Seoul 01704, Republic of Korea

**Keywords:** extraskeletal osteosarcoma, mineralization, peripheral rim enhancement, metastasis, computed tomography

## Abstract

**Simple Summary:**

Extraskeletal osteosarcoma is an osteoid-producing neoplasm without primary bone involvement. Imaging findings of extraskeletal osteosarcoma are well described in human medicine; however, while there have been several case reports and studies of canine extraskeletal osteosarcoma addressed in veterinary medicine, there is limited information on the computed tomography findings of the disease. Therefore, the goal of the present case series was to investigate the computed tomography characteristics of extraskeletal osteosarcoma in dogs.

**Abstract:**

The objective of the present case series was to investigate the various computed tomography findings of six dogs diagnosed with extraskeletal osteosarcoma (exOSA) at several locations. Among the tumors evaluated, four were subcutaneous, one was mammary, and one involved the intestinal tract. Intralesional mineralization was observed in all six dogs. Most of the tumors were moderately calcified, exhibited amorphous mineralization, and were heterogeneous on post-contrast imaging. Three of the tumors were peripherally enhanced, and regional lymphadenopathy was identified in two of the dogs, which was presumed to be metastatic. No lymph node calcification was reported. Although the presence of intralesional mineralization is not a pathognomonic finding, it was consistently identified in the present case series. Therefore, exOSA should be considered in the differential diagnosis when mineralization occurs in a mass unrelated to osseous structures.

## 1. Introduction

Osteosarcoma, the most frequent primary bone tumor in dogs, is a malignant mesenchymal neoplasm characterized by the production of osteoid or immature bone cells and affects the appendicular skeleton 3–4 times more frequently than the axial skeleton [1,2,3]. Furthermore, the tumor is classified into five subtypes—central, surface (juxtacortical), parosteal, periosteal, and extraskeletal—with central being the most common [4]. Central osteosarcoma arises within the bones, is considered more malignant than periosteal or parosteal tumors, and is further categorized into the following subtypes: osteoblastic, chondroblastic, fibroblastic, telangiectatic, and giant cell-rich osteosarcoma [4]. Parosteal osteosarcoma arises from the outer fibrous layer of the periosteum; however, it is poorly described in veterinary medicine [4,5]. Periosteal osteosarcoma, which originates from undifferentiated mesenchymal cells in the cambium layer of the periosteum, has also been rarely described [4]. Extraskeletal osteosarcoma (exOSA) is an osteoid-producing neoplasm without primary bone involvement [4]. Accounting for 1% of all osteosarcoma cases, exOSA can occur in a variety of organs [6]. Cases of canine exOSA involving the gastrointestinal tract, subcutaneous tissue, spleen, urinary tract, liver, mediastinum, skin, muscle, eyes, central nervous system, omentum, pericardium, and thyroid gland have been reported [6,7,8,9,10,11,12,13,14,15]. Additionally, exOSA tends to affect older dogs more frequently than skeletal osteosarcoma, although no predispositions regarding dog size have been reported [6,7]. While skeletal osteosarcoma usually occurs in dogs weighing > 15 kg, cases of exOSA have been reported in dogs weighing < 15 kg [9,16]. When treated with surgery alone, exOSA resulted in a shorter median survival time (25 days) than skeletal osteosarcoma (132–154 days) [2,17]. The median survival time increases when patients receive chemotherapy [8]. In one study, the dogs that received chemotherapy had a mean survival time of 146 days, compared to 33 days for those that did not [8].

The imaging findings of skeletal osteosarcoma have been well addressed in the veterinary literature. Bone destruction, periosteal reactions, cortical erosion, expansion or penetration, and the presence of adjacent soft tissue masses are the key radiographic findings to help differentiate benign and malignant bone tumors [4]. Although not always present, Codman’s triangle and sunburst periosteal reactions have been described in osteosarcoma and other malignant bone tumors [4]. Similarly, the computed tomography (CT) findings of osteosarcoma and other malignant tumors arising from the bones include cortical bone destruction, amorphous periosteal reactions, and ill-defined transition zones [18].

The CT findings of exOSA in humans have been well reported and include mineralization, pseudocapsule, and heterogeneous enhancement [19]. However, there is a paucity of research describing the imaging findings of exOSA in veterinary medicine. Radiographic findings of intestinal exOSA, magnetic resonance imaging (MRI) findings of intradural exOSA, positron emission tomography findings of injection-induced exOSA, and CT findings of salivary gland, kidney, and post-hepatic caudal vena cava exOSA have been reported in individual cases [2,17,18,19,20,21,22,23,24]. As of yet, however, no studies have investigated the spectrum of CT findings of exOSA among multiple dogs, which therefore is what we aimed to investigate in the present study.

## 2. Material and Methods

### 2.1. Ethics Statement

Due to the retrospective nature of the present study, ethical review and approval were not required. However, written informed consent for publication was obtained from the patients’ owners.

### 2.2. Study Design

Five centers participated in the present retrospective, multicenter case series: Konkuk Veterinary Teaching Hospital, Shine Animal Medical Center, VIP Animal Medical Center, Ilsan Animal Medical Center, and Nowon N Animal Medical Center. We screened their respective databases for cases of exOSA from 2016 to 2023. The inclusion criteria were as follows: CT images available for review, evidence of a mass arising from an extraskeletal region, and a histopathological diagnosis of osteosarcoma. Cases were excluded if there was equivocal evidence of the involvement of skeletal structures (e.g., presence of periosteal reaction). Biopsy specimens or whole mass excisions performed by veterinary surgeons were used for the histopathological analysis and subsequent interpretation by veterinary pathologists.

### 2.3. Data Collection, CT Examinations, and Image Analysis

The following data were obtained from the patient medical records of each institution: breed, sex, age, body weight, anamnesis, clinical examination, surgical interventions, and chemotherapy treatment. The CT examinations were performed using a variety of scanners, ranging from 4 to 64 slices, including the Alexion (Canon Medical Corporation, Tochigi, Japan), Aquilion (Canon Medical Corporation, Tochigi, Japan), Revolution ACT (General Electric Medical System, Chicago, IL, USA), Lightspeed (General Electric Medical System, Chicago, IL, USA), and Emotion 16 (Siemens Healthcare, Erlangen, Germany). The CT acquisition settings included the following: slice thickness, 0.75–3 mm; helical pitch, 0.75–3; matrix dimension, 512 × 512; and field of view, variable. The specific image acquisition parameters and number of scans performed are summarized in Table 1. The CT procedure was performed as follows: patients were placed in either the supine or prone position under general anesthesia; a nonionic contrast medium (iohexol 350 mg/mL [Omnipaque]; GE Healthcare, Princeton, NJ, USA) was administered manually or by a power injector at a rate of 2–2.5 mL/s; post-contrast images were acquired 70–80 s after the administration of contrast; and no arterial phase images were obtained. The CT images were displayed with three window settings—soft tissue window (window level, 60; window width, 400), bone window (window level, 300; window width, 1500), and lung window (window level, −400; window width, 1500). Both pre- and post-contrast images were evaluated. The images were reviewed using commercially available image viewing software (Radiant; Medixant, Proznan, Poland) by a clinician with 4 years of experience in veterinary diagnostic imaging (JJ) under the supervision of a senior clinician with >10 years of experience in radiology (JK).

The following qualitative CT features were assessed: location (subcutaneous, mammary, or visceral), margins (ill- or well-defined), enhancement pattern (homogeneous or heterogeneous, presence or absence of peripheral rim enhancement), mineralization (present or absent), degree of mineralization (mild, moderate, or marked), shape of mineralization (dot, spindle, or amorphous), location of mineralization (concentric, eccentric, or mixed within the tumor), presumptive pulmonary metastasis (present or absent), and lymphadenopathy (present or absent) [25]. Mineralization was assessed using a bone window, whereas other features were evaluated with a soft tissue window, except for the presence of pulmonary metastasis, which was assessed using a lung window. Lymphadenopathy was considered when lymph nodes showed significant enlargement with abnormal shape and/or attenuation. The quantitative CT features assessed included tumor length, width, and height.

## 3. Results

### 3.1. Patient Description

**Figure 1:**
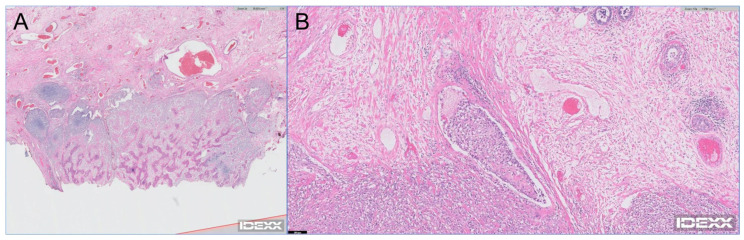
Histopathological images of dog 1 (**A**) and dog 5 (**B**). Chondroblastic osteosarcoma along with soft tissue and focal cartilage remodeling, proliferative new bone, and fibrosis are observed (**A**). Mammary osteosarcoma, arranged in highly cellular lobules that multifocally form the basophilic chondroid and eosinophilic osteoid matrix, is observed (**B**).

A total of six spayed female dogs (Golden retriever, English cocker spaniel, Maltese, Schnauzer, Welsh corgi, and Great Pyrenees) were included in the present study. The median age and body weight were 11.7 (range, 7.0–14.7) years and 12.0 (1.9–45.0) kg, respectively. Five of the dogs were brought in due to the presence of a palpable mass, while one was brought in for diarrhea. Excisional biopsies were performed in five cases, while an incisional biopsy was performed in one case (Figure 1).

### 3.2. CT Features

**Figure 2:**
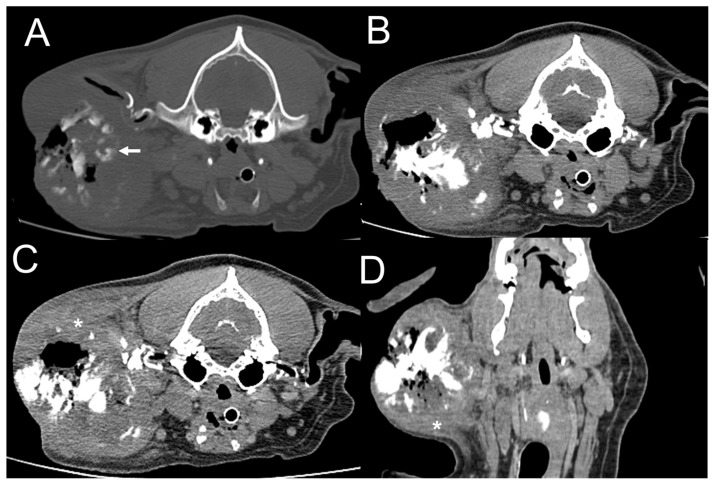
Dog 1. Transverse plane, pre-contrast computed tomography (CT) images with bone (**A**) and soft tissue (**B**) windows and post-contrast CT image with a soft tissue window (**C**). Dorsal plane, post-contrast CT image with a soft tissue window (**D**). A subcutaneous mass invades the right parotid region, showing marked, amorphous, and mixed mineralization (arrow). The mass is ill-defined and shows heterogeneous contrast enhancement (asterisk). There is no peripheral rim enhancement.

**Figure 3:**
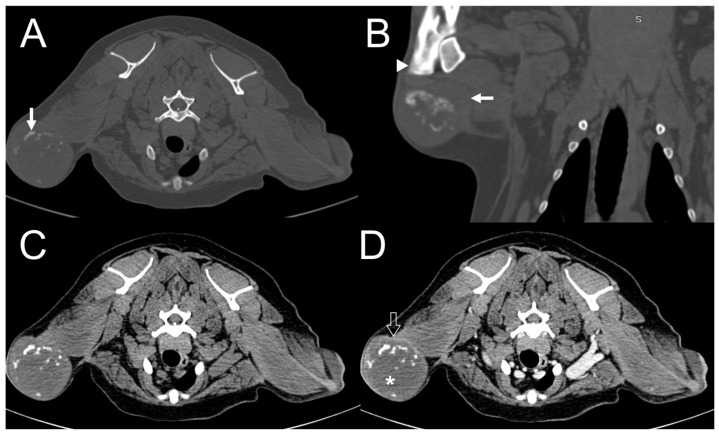
Dog 2. Transverse (**A**) and dorsal (**B**) plane pre-contrast computed tomography (CT) images with a bone window. Transverse plane pre- (**C**) and post-contrast (**D**) CT images with a soft tissue window. A well-defined subcutaneous mass invading the right forelimb shows moderate, spindle-shaped, and mixed mineralization (arrow). The adjacent bones (arrowhead) are not involved. The center of the lesion does not show contrast uptake (asterisk), although peripheral rim enhancement is present (open arrow).

**Figure 4:**
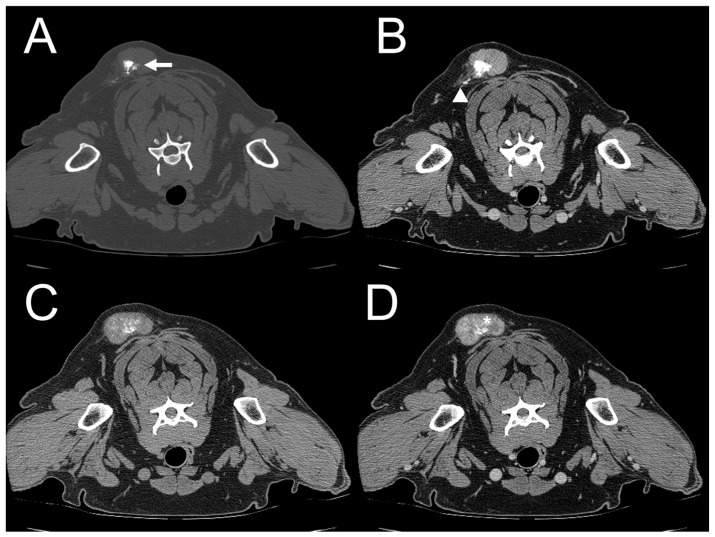
Dog 3. Transverse plane pre-contrast computed tomography (CT) images with bone (**A**) and soft tissue (**C**) windows and post-contrast CT images with a soft tissue window (**B**,**D**). An ill-defined subcutaneous mass invading the dorsum at the level of C4–5 shows moderate, amorphous, and mixed mineralization (arrow). The mass is heterogeneous on post-contrast images and does not exhibit peripheral rim enhancement (asterisk).

**Figure 5:**
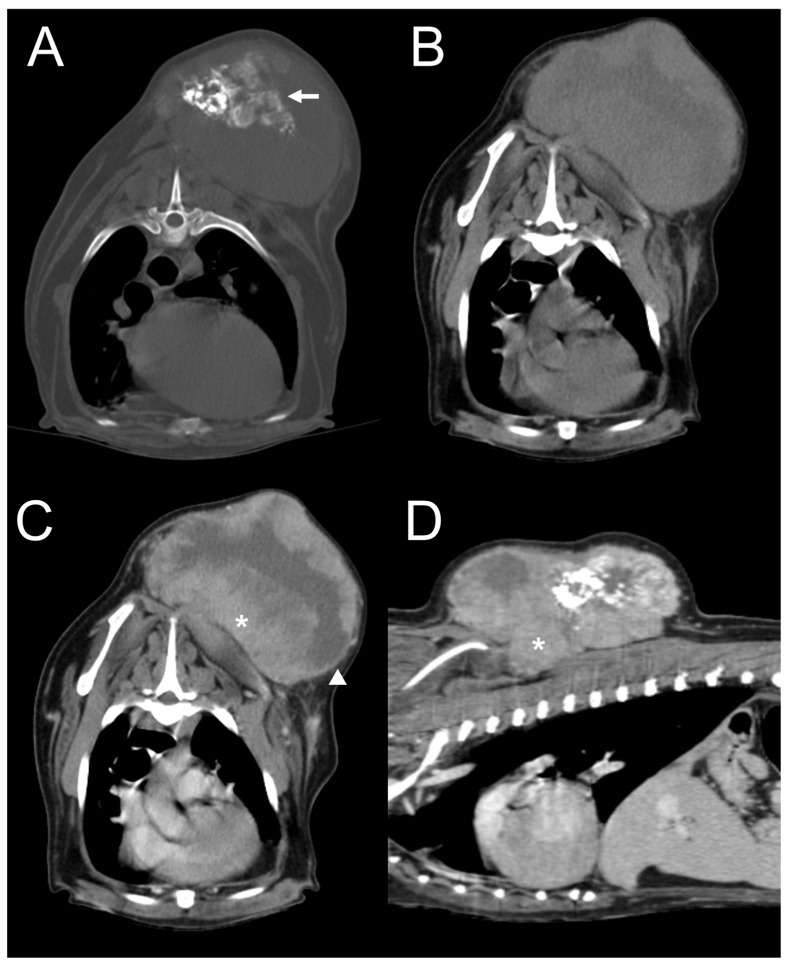
Dog 4. Transverse plane pre-contrast computed tomography (CT) images with bone (**A**) and soft tissue (**B**) windows and post-contrast CT image with a soft tissue window (**C**). Sagittal plane post-contrast CT image with a soft tissue window (**D**). A well-defined subcutaneous mass invading the dorsum at the level of T1–8 shows moderate, amorphous, and mixed mineralization (arrow). The mass shows heterogenous (asterisk) and peripheral rim (arrowhead) enhancement.

**Figure 6:**
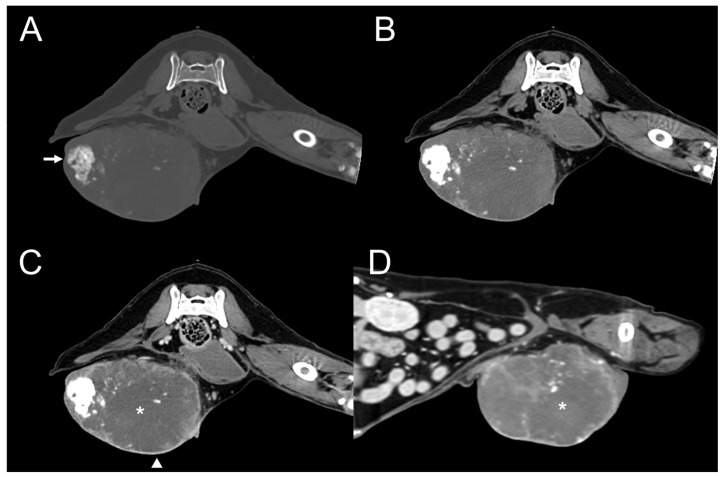
Dog 5. Transverse plane pre-contrast computed tomography (CT) images with bone (**A**) and soft tissue (**B**) windows and post-contrast CT image with a soft tissue window (**C**). Sagittal plane post-contrast CT image with a soft tissue window (**D**). A well-defined mass invading the right caudal mammary region shows moderate, amorphous, and eccentric mineralization (arrow). The mass shows heterogenous (asterisk) and peripheral rim (arrowhead) enhancement.

**Figure 7:**
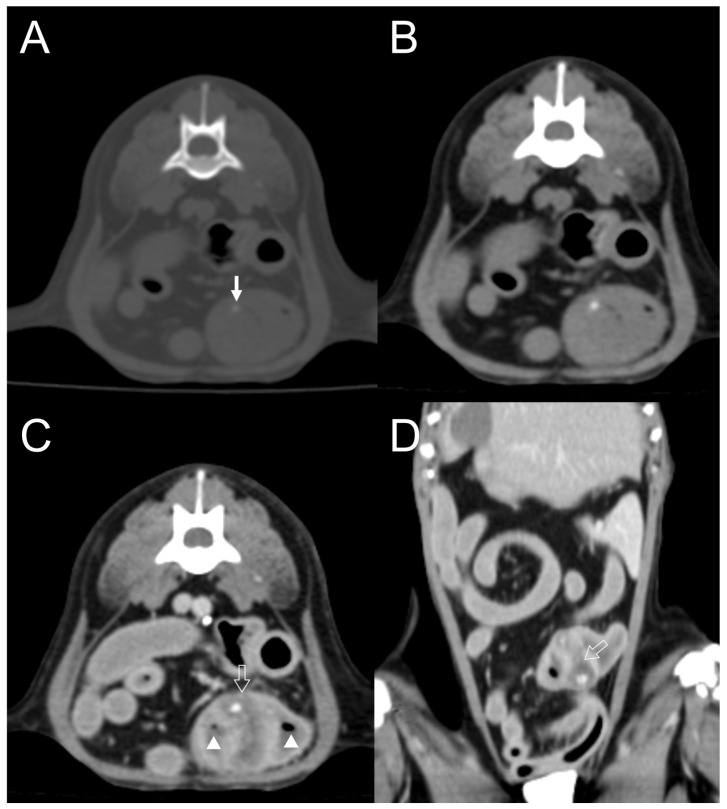
Transverse plane, pre-contrast computed tomography (CT) images with bone (**A**) and soft tissue (**B**) windows and post-contrast CT image with a soft tissue window (**C**). Dorsal plane, post-contrast CT image with a soft tissue window (**D**). A well-defined mass invading the small intestine shows mild, dot-shaped, and eccentric mineralization (arrow). The mass homogeneously uptakes the contrast (open arrow). Because the mass is surrounded by the contrast-enhanced intestinal wall, peripheral rim enhancement is considered absent. Gas bubbles (arrowhead) are present within the intestinal lumen, adjacent to the mass.

The location of each exOSA varied as follows: subcutaneous, right parotid (Figure 2), right forelimb (Figure 3), dorsum at the level of the 4th–5th cervical vertebrae (C4–5) (Figure 4), dorsum at the level of the 1st–8th thoracic vertebrae (T1–8) (Figure 5), right caudal mammary (Figure 6), and small intestine (Figure 7). The median length, width, and height of the tumors were 7.0 (range, 2.1–11.5) cm, 5.3 (range, 1.6–9.4) cm, and 6.8 (1.3–11.4) cm, respectively.

The CT features of exOSA were evaluated as seen in Table 2. Of the six dogs with exOSA, two (33.3%) had ill-defined margins, while the other four (66.7%) exhibited well-defined margins; four (66.7%) had heterogeneous while one had homogeneous enhancement on post-contrast images, and one had no core enhancement; three (50%) exhibited peripheral rim enhancement, while the other three (50%) did not; mineralization was seen in all six tumors (100%); one (16.7%) showed mild, four (66.7%) moderate, and one (16.7%) marked mineralization; one (16.7%) showed dot-shaped and one (16.75) showed spindle-shaped mineralization, while the other four tumors (66.7%) exhibited amorphous mineralization; the location of mineralization was eccentric in two tumors (33.3%) and mixed in four (66.7%), while no concentric tumor mineralization was observed.

**Figure 8:**
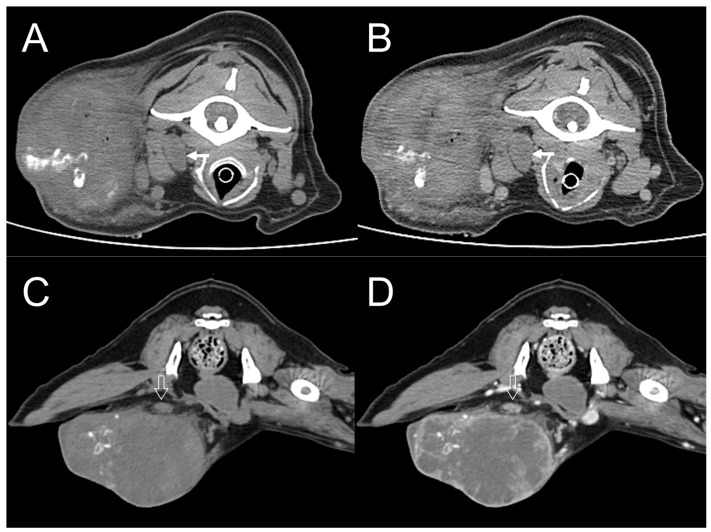
Dogs 1 and 5. Transverse plane, pre- (**A**,**C**) and post-contrast (**B**,**D**) computed tomography (CT) images with a soft tissue window. In dog 1, the right medial retropharyngeal lymph node is enlarged, and minimal contrast enhancement is seen (arrow) (**A**,**B**). In dog 5, the right superficial inguinal lymph node is enlarged and ovoid and shows heterogeneous contrast enhancement (open arrow) (**C**,**D**).

Lymphadenopathy was detected in two of the cases. Dog 1 had a subcutaneous tumor in the right parotid region, which showed right medial retropharyngeal lymphadenopathy (Figure 8A,B). Dog 5 had a tumor in the right caudal mammary region, which exhibited right superficial inguinal lymphadenopathy (Figure 8C,D). Lymph node mineralization was not appreciated in either case, nor was a histopathological examination of the suspicious lymph nodes performed. No pulmonary nodules were identified.

### 3.3. Follow-up

After the initial CT examinations, the treatment courses were as follows: dogs 1, 2, 4, and 6 underwent mass excision, while dog 5 underwent bilateral mastectomy, and dog 3 underwent an incisional biopsy with no further treatment. Additionally, dog 2 received intravenous carboplatin at a dose of 300 mg/m^2^ × 4 times in 3-week intervals, dog 4 received toceranib at a dose of 3 mg/kg every other day for 2 months, and dogs 1 and 5 did not receive chemotherapy.

**Figure 9:**
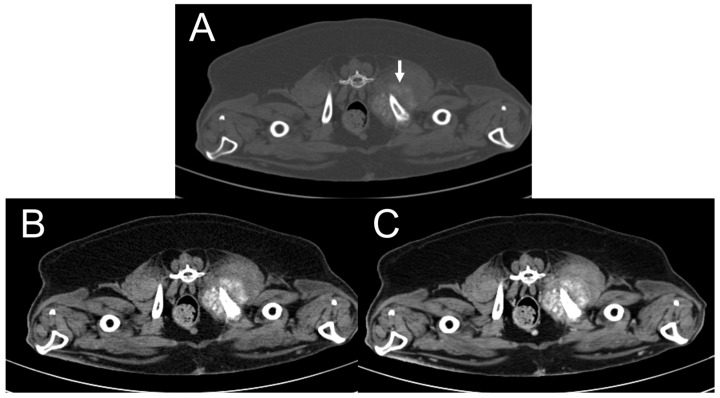
Dog 2. Follow-up computed tomography (CT) 9 months after the initial CT examination. Transverse plane, pre-contrast CT image with bone (**A**) and soft tissue (**B**) windows and post-contrast CT image with a soft tissue window (**C**). There is a hyperattenuating, heterogeneous, and non-contrast-enhancing mass centered on the left ilium. An amorphous periosteal reaction is present (arrow).

A follow-up CT was available for dog 2, who was brought in due to the development of a new mass in the left pelvic region 9 months after the initial excision. The CT examination showed a periosteal reaction of the left ilium (Figure 9); therefore, the differential diagnoses included primary bone osteosarcoma, chondrosarcoma, other soft tissue sarcoma with secondary bony involvement, and exOSA metastasis. There was no evidence of local recurrence at the previous excision site, and no histopathological diagnosis was obtained. Since bone was affected and no final diagnosis was reached, this follow-up examination was not included in the present study.

## 4. Discussion

The present case series aimed to describe the spectrum CT features observed in a group of dogs diagnosed with exOSA in various regions of the body. The included dogs had a median age of 11.7 years, no breed predominance was observed, and all of the dogs were female, similar to the findings of previous veterinary studies [6,8]. The median body weight of the dogs in the present study, however, was slightly lower than that observed in previous studies [6,8].

ExOSA has been reported to occur in a variety of locations [6,7,8,9,10,11,12]. Langenbach et al. [7] divided dogs diagnosed with exOSA into the following two groups, based on origin: soft tissue and mammary gland. In their study, 36% of the exOSA tumors originated from the soft tissue, while the remaining 64% originated in the mammary glands. Within the soft tissue group, gastrointestinal, subcutaneous, and splenic origins were the most common, and within the mammary group, the fourth and fifth mammary glands were the most common origins [7]. In the present study, most of the exOSA tumors were located in the subcutaneous region, with only one involving the mammary tissue; however, only six cases were included in the present study, which could explain the discrepancy with the aforementioned publication.

The imaging findings of exOSA are well described in human medicine, with both CT and MRI commonly used to diagnose and assess the lesions. Common CT findings include a variable degree of mineralization, the presence of pseudocapsules, and heterogeneous contrast enhancement. Although tumor calcification is not systematic, it occurs in approximately 50% of the cases [19].

In the present case series, all of the exOSA tumors were calcified, with the tumor mineralizations mostly described as moderate, with an amorphous shape and mixed location in half of the cases. These results are contrary to the veterinary study by Kuntz et al. [8] in which only 31% of the exOSA tumors were mineralized. However, because their study was based on radiographic findings, small degrees of tumor calcification could have been overlooked, while the present study was based on CT findings, which is superior in the detection of subtle mineralization or osteoid matrices. This difference, therefore, may have contributed to the higher prevalence of tumor calcification between the two studies. In a study of human cases, it was reported that 62% of exOSA tumors were calcified, as follows: mineralization extent <10% of tumor volume in 12/42 patients, 10–50% in 7/42 patients, and >50% in 7/42 patients. Amorphous calcification was the most common pattern (18/26), followed by spindle-shaped (6/26) and dot-shaped (2/26) mineralization [25]. In comparison, the present study demonstrated a higher prevalence of tumor mineralization. Most of the tumors evaluated in the present case series were moderately calcified; however, this criterion is subjective, which makes an accurate comparison with the aforementioned study challenging. However, in accordance with the aforementioned study, amorphous calcifications were the most commonly reported tumor mineralization shapes in the present study.

Most of the exOSA tumors in the present study showed heterogeneous contrast enhancement, while half of them displayed peripheral rim enhancement. Heterogeneous enhancement may be a common CT finding of exOSA, although further investigations are needed. The three tumors with peripheral rim enhancement showed well-defined margins, while two of the tumors without peripheral rim enhancement were ill defined, and one involved the intestinal tract and was considered to be well defined due to the presence of the intestinal wall surrounding it. In humans, heterogeneous enhancement seems to be a typical finding in exOSA, and one study based on MRI reported that no exOSA tumors showed homogeneous contrast enhancement [19,26]. Although the imaging modalities differed, one dog in the present study with an exOSA tumor invading the intestinal tract showed homogeneous enhancement. The enhancement pattern may differ if the exOSA tumor originates from a visceral organ; however, further studies are needed to evaluate this difference.

The presence of intralesional mineralization, heterogenous contrast enhancement, and peripheral rim enhancement were common CT features in the present case series; however, many other tumor types show similar characteristics. In the study by Furest et al. [26], 81% of liposarcomas were heterogeneous, 62% were encapsulated, and 23% demonstrated intralesional calcifications. In another study, undifferentiated pleomorphic sarcomas were described as large, lobulated heterogenous masses with contrast-enhanced septa, although no intralesional mineralization was observed [27]. In the study by Farmer et al. [28], most subcutaneous, intramuscular, and intermuscular mast cell tumors (MCTs) showed heterogenous enhancement (62%). The presence or absence of intralesional mineralization was not investigated in the aforementioned study; however, in the authors’ experience, mineralization seems to be uncommon in MCTs. In a study investigating the appearance of nonparenchymal hemangiosarcomas, only 1 tumor (1/17) was mineralized, while ring enhancement was observed in 4/17 cases [29].

Tumor calcification is not an exclusive feature of exOSA. In fact, osseous osteosarcomas are almost always mineralized. However, in the study by Cordella et al. [30], most of the osseous osteosarcomas showed centrally located calcifications, while in the present study, no exOSA tumors exhibited central mineralization. Additionally, osseous osteosarcomas showed a higher prevalence of lymphadenopathy and presumed pulmonary metastasis in the aforementioned study than in the present case series [30]. These features may, for example, help differentiate osseous osteosarcoma from exOSA tumors when a mass is close to a bone; however, due to the small number of cases in the present study, further research is needed.

In human medicine, metastatic exOSA lesions may or may not show mineralization [19]. Mineralization was not present in the presumed metastatic lymph nodes in the present case series. Previous veterinary studies have shown conflicting results regarding the prevalence of metastasis associated with exOSA in various organs, including lung, liver, kidney, heart, omentum, and mediastinum [8,31]. Kuntz et al. [8] reported that 85% of dogs presented with metastasis by the time of death, whereas Duffy et al. [31] reported that only 21% of dogs were diagnosed with metastasis. Two of the dogs in the present study were suspected of having metastatic spread into a lymph node.

In dog 2 of the present case series, a calcified lesion was identified within the left ilium 9 months after the diagnosis of exOSA in a different body region. Primary bone tumors and metastases of exOSA to the bone were both considered in that case, although no definitive diagnosis was reached. In humans, bone metastases were reported in 8–19% of exOSA cases [19]. To the best of the authors’ knowledge, however, no cases of bone metastases associated with exOSA in veterinary medicine have been published to date.

The present study had several limitations, including the small number of cases, the retrospective nature of the study, the non-standardized CT protocols among the different institutions, the absence of a histopathological confirmation of nodal metastasis, and the absence of correlation between the CT and histopathological features. Correlating the histopathological evaluation of the mineralized lesions to the CT findings would have enhanced the clinical significance of the present case series. Despite the long periods of observation in the present case series, a small number of cases were included since we required both histopathological and CT examinations. Additionally, if a mass showed a periosteal reaction in adjacent bones, that case was excluded due to the possibility that skeletal osteosarcoma could not be completely ruled out. The tight inclusion criteria of the present study were necessary to ensure an accurate diagnosis of exOSA; however, this resulted in a very limited number of cases being included.

## 5. Conclusions

The present case series reported the CT features of exOSA in six dogs. Intralesional mineralization and heterogeneous contrast enhancement were common CT features, while peripheral rim enhancement was only detected in some of the cases. Although the presence of intralesional calcification is not a pathognomonic feature of exOSA, this diagnosis should be considered in the differential diagnosis of cases presenting with mineralized masses unrelated to the skeleton.

## Figures and Tables

**Table 1:** Summary of imaging acquisition parameters and number of scans performed.

	Manufacturer	Model	CT Channel	Slice Thickness	Helical Pitch	Matrix Dimension	kVp	mAs	Number of Scans Performed
Dog 1	Toshiba	Alexion	16	3	0.9	512	100	120	1
Dog 2	Siemens	Emotion 16	16	0.75	0.8	512	130	134	2 *
Dog 3	Toshiba	Aquilion 64	64	1	N/A	512	120	150	1
Dog 4	GE	LightSpeed	4	2.5	1.5	512	120	200	1
Dog 5	Toshiba	Alexion	16	3	0.9	512	120	200	1
Dog 6	GE	Revolution ACT	32	2.5	0.5	512	120	80	1

CT, computed tomography; kVp, kilovoltage peak; mAs, milliampere-seconds; N/A, not available. * Follow-up CT was performed but not included in the present study.

**Table 2:** Summary of CT features for each case.

	Location	Margins	Enhancement		Mineralization			
			Homogeneity	Peripheral Rim Enhancement	Presence	Degree	Shape	Location
Dog 1	Subcutaneous (right parotid)	Ill-defined	Heterogenous	Absent	Present	Marked	Amorphous	Mixed
Dog 2	Subcutaneous (right forelimb)	Well-defined	Absent	Present	Present	Moderate	Spindle	Mixed
Dog 3	Subcutaneous (dorsum at level of the 4th–5th cervical vertebrae (C4–5))	Ill-defined	Heterogenous	Absent	Present	Moderate	Amorphous	Mixed
Dog 4	Subcutaneous (dorsum at the level of the 1st–8th thoracic vertebrae (T1–8))	Well-defined	Heterogeneous	Present	Present	Moderate	Amorphous	Mixed
Dog 5	Mammary (rt. caudal)	Well-defined	Heterogenous	Present	Present	Moderate	Amorphous	Eccentric
Dog 6	Small intestine	Well-defined	Homogenous	Absent	Present	Mild	Dot	Eccentric

## Data Availability

The data from the present study are available from the corresponding author upon reasonable request.

## References

[1] Brodey R.S., Riser W.H. (1969). Canine osteosarcoma: A clinicopathologic study of 194 cases. Clin. Orthop. Relat. Res..

[2] Spodnick G.J., Berg J., Rand W.M., Schelling S.H., Couto G., Harvey H.J., Henderson R.A., MacEwen G., Mauldin N., McCaw D.L. (1992). Prognosis for dogs with appendicular osteosarcoma treated by amputation alone: 162 cases (1978–1988). J. Am. Vet. Med. Assoc..

[3] Wolke R.E., Nielsen S.W. (1966). Site incidence of canine osteosarcoma. J. Small Anim. Pract..

[4] Thompson K.G., Dittmer K.E., Meuten D.J. (2016). Tumors of bone. Tumors in Domestic Animals.

[5] Banks W.C. (1971). Parosteal osteosarcoma in a dog and a cat. J. Am. Vet. Med. Assoc..

[6] Patnaik A.K. (1990). Canine extraskeletal osteosarcoma and chondrosarcoma: A clinicopathologic study of 14 cases. Vet. Pathol..

[7] Langenbach A., Anderson M.A., Dambach D.M., Sorenmo K.U., Shofer F.D. (1998). Extraskeletal osteosarcomas in dogs: A retrospective study of 169 cases (1986–1996). J. Am. Anim. Hosp. Assoc..

[8] Kuntz C.A., Dernell W.S., Powers B.E., Withrow S. (1998). Extraskeletal osteosarcomas in dogs: 14 cases. J. Am. Anim. Hosp. Assoc..

[9] Rezende Souza F., de Morais Avelar N., Moreira Lopes T.C., Dantas Cassali G., Yumi Ribeiro Nakagaki K. (2023). Extraskeletal osteosarcoma in the duodenum of a dog. Acta Sci. Vet..

[10] Alberti T.D., Zamboni R., Venancio F.D., Brunner C.B., Raffi M.B., Schild A.L., Sallis E.S. (2021). Mediastinal extraskeletal osteosarcoma in a canine with pulmonary and cerebral metastasis. Acta Sci. Vet..

[11] Johnson C., Kim Y. (2013). Hepatic extraskeletal osteosarcoma with systemic metastasis in a dog. Korean J. Vet. Res..

[12] Miller M.A., Aper R.L., Fauber A., Blevins W.E., Ramos-Vara J.A. (2006). Extraskeletal osteosarcoma associated with retained surgical sponge in a dog. J. Vet. Diagn. Investig..

[13] Soares C.T., Medeiros F.P., Martins R. (2023). Primary omentum extraskeletal osteosarcoma in a dog: Case report. Braz. J. Vet. Med..

[14] Sato T., Koie H., Shibuya H., Suzuki K. (2004). Extraskeletal osteosarcoma in the pericardium of a dog. Vet. Rec..

[15] Riggers D.S., Rosati M., Köhler C., Matiasek K., Loderstedt S. (2022). A case of extraosseous intradural osteosarcoma of the spine in a dog. Vet. Rec..

[16] Kistler K.R. (1981). Canine osteosarcoma: 1,462 cases reviewed to uncover patterns of height, weight, breed, sex, age and site of involvement. Phi Zeta Awards.

[17] Heyman S.J., Diefenderfer D.L., Goldschmidt M.H., Newton C.D. (1992). Canine axial skeletal osteosarcoma. A retrospective study of 116 cases (1986 to 1989). Vet. Surg..

[18] Schultz R.M., Wisner E.R., Tobias S., Jimmy S. (2011). Long bones. Veterinary Computed Tomography.

[19] Mc Auley G., Jagannathan J., O’Regan K., Krajewski K.M., Hornick J.L., Butrynski J., Ramaiya N. (2012). Extraskeletal osteosarcoma: Spectrum of imaging findings. AJR Am. J. Roentgenol..

[20] MacKenzie S., Hecht S., Sura P.A., Craig L.E. (2012). What is your diagnosis? Extraskeletal osteosarcoma. J. Am. Vet. Med. Assoc..

[21] Selmic L.E., Griffin L.R., Rector M.H., Lafferty M., Pool R., Ehrhart N.P. (2016). Treatment of extraskeletal osteosarcoma at a previous injection site resulting in prolonged survival in 1 dog. Can. Vet. J..

[22] Umeda N., Yamazoe H., Wada A., Nagata K. (2023). A dog with extraskeletal osteosarcoma of the salivary glands survived long-term, following surgical resection and adjuvant therapy. J. Vet. Med. Sci..

[23] Garcia M.R., Gomes B., Irvine K., Rosa C. (2023). Metastatic renal extraskeletal osteosarcoma in a dog: Clinical presentation, CT features and the role of ALP cytochemical staining for diagnosis. Vet. Rec..

[24] Tremolada G., Griffin L., Manchester A.C., Aboellail T., Lapsley J.M., Selmic L.E. (2023). Primary extraskeletal osteosarcoma of the post-hepatic caudal vena cava in a dog-Case report. Front. Vet. Sci..

[25] Crombé A., Spinnato P., Righi A., Leopardi M.P., Carpenzano M., Izzo F., Parmeggiani A., Linck P.A., Perret R., Cesari M. (2023). Imaging presentation of extraskeletal osteosarcomas on CT and MRI and correlation with patients outcome: A two-center retrospective of 54 patients. Diagn. Interv. Imaging.

[26] Fuerst J.A., Reichle J.K., Szabo D., Cohen E.B., Biller D.S., Goggin J.M., Griffin J.F., Aarsvold S., Emerson S.E. (2017). Computed tomographic findings in 24 dogs with liposarcoma. Vet. Radiol. Ultrasound.

[27] Choi H., Kwon Y., Chang J., Jeong S., Lee H., Kim J., Jung J., Lee Y. (2011). Undifferentiated pleomorphic sarcoma (malignant fibrous histiocytoma) of the head in a dog. J. Vet. Med. Sci..

[28] Farmer R.J.M., Poirier V.J., Nykamp S., Jensen M., Foster R.A., Oblak M., Appleby R. (2023). CT features of subcutaneous, intermuscular, and intramuscular mast cell tumors in dogs. Vet. Radiol. Ultrasound.

[29] Fukuda S., Kobayashi T., Robertson I.D., Oshima F., Fukazawa E., Nakano Y., Ono S., Thrall D.E. (2014). Computed tomographic features of canine nonparenchymal hemangiosarcoma. Vet. Radiol. Ultrasound.

[30] Cordella A., Stock E., Bertolini G., Strohmayer C., Serra G.D., Saunders J. (2023). CT features of primary bone neoplasia of the thoracic wall in dogs. Vet. Radiol. Ultrasound.

[31] Duffy D., Selmic L.E., Kendall A.R., Powers B.E. (2017). Outcome following treatment of soft tissue and visceral extraskeletal osteosarcoma in 33 dogs: 2008–2013. Vet. Comp. Oncol..

